# Early Somatosensory Processing Over Time in Individuals at Risk to Develop Psychosis

**DOI:** 10.3389/fpsyt.2019.00047

**Published:** 2019-02-13

**Authors:** Florence Hagenmuller, Karsten Heekeren, Patrik Roser, Helene Haker, Anastasia Theodoridou, Susanne Walitza, Wulf Rössler, Wolfram Kawohl

**Affiliations:** ^1^The Zurich Program for Sustainable Development of Mental Health Services (ZInEP), University of Zurich, Zurich, Switzerland; ^2^Department of Psychiatry, Psychotherapy and Psychosomatics, University of Zurich, Zurich, Switzerland; ^3^Department of Psychiatry and Psychotherapy, Psychiatric Services Aargau, Academic Hospital of the University of Zurich, Brugg, Switzerland; ^4^Translational Neuromodeling Unit, Institute for Biomedical Engineering, University of Zurich and ETH Zurich, Zurich, Switzerland; ^5^Department of Child and Adolescent Psychiatry, University of Zurich, Zurich, Switzerland; ^6^Laboratory of Neuroscience (LIM 27), Institute of Psychiatry, University of São Paulo, São Paulo, Brazil; ^7^Department of Psychiatry and Psychotherapy, Charité University Medicine, Berlin, Germany

**Keywords:** somatosensory processing, SEP, thalamus, psychosis, at-risk, transition, cannabis use

## Abstract

**Objective:** Somatosensory evoked potentials (SEPs) enable the investigation of thalamocortical and early cortical processing. Previous studies reported alterations of SEPs in patients with schizophrenia as well as in individuals in the prodromal stage. Moreover, cannabis use as an environmental risk factor for the development of schizophrenia has been demonstrated to influence SEP parameters in individuals at risk to develop psychosis. The aim of this study was to explore the course of SEP changes and the impact of concomitant cannabis use in individuals at risk to develop psychosis who sought medical help.

**Methods:** Median nerve SEPs including high-frequency oscillations (HFOs) superimposed on the primary cortical response (N20) were investigated using multichannel EEG in individuals (*n* = 54 at baseline) remaining at risk to develop psychosis at follow-up after 1 year (high-risk: *n* = 19; ultra-high-risk: *n* = 27) vs. subjects with conversion to psychosis (*n* = 8) and a healthy control group (*n* = 35). Longitudinal and cross-sectional analyses of SEP components as estimated by dipole source analysis were performed.

**Results:** The longitudinal development of the N20 strength depended on cannabis use. In cannabis non-users, a greater decrease of N20 strengths over time was associated with more negative symptoms at baseline. At baseline, converters did not differ from subjects remaining at risk. At follow-up, converters showed increased low- and high-frequency activity than at-risk subjects and did not differ from controls.

**Conclusion:** The results of this study lead to the suggestion that the deficits in early somatosensory processing in individuals at risk to develop psychosis may not represent a marker for a genetic risk for psychosis but rather reflect state-dependent factors such as negative symptoms. On the other hand, the transition to psychosis seems to represent an interstage between reduced sensory registration from the at-risk state and gating deficits in the chronic state.

## Introduction

The neuropathological picture of psychosis is not static but changes over time (1). Patients with psychosis often show less severe manifestations of the illness for days up to years prior to onset of full-blown clinical picture (2). These early signs are characterized by subtle, self-experienced deficits and mostly non-specific symptoms, also termed “basic symptoms” (3) often accompanied by attenuated negative symptoms (3–5). Early signs that are more proximal to the onset of psychosis tend to be more specific for psychotic disorders and are mostly characterized by attenuated positive symptoms of psychosis (6, 7). The term “psychosis risk syndrome” was proposed to indicate a possible risk of developing psychosis (4, 8).

Early intervention is thought to reduce the duration of untreated psychosis and to prevent or delay transition to psychosis by attenuating the symptoms of individuals at clinical high-risk for psychosis (4, 9). Higher levels of attenuated positive symptoms, poor social functioning, some genetic risk, e.g., having a first-degree relative with a psychotic disorder, have been so far identified as consistent predictors of conversion to psychosis (10). There is additionally some evidence that cannabis use, respectively cannabis use on top of a genetic vulnerability, increases the risk of developing psychosis (11–13).

For better prognostic accuracy, neurobiological markers are also increasingly used in early recognition of psychosis. Human median nerve somatosensory evoked potentials (SEPs) offer the opportunity to investigate thalamocortical and early cortical processing (14–17). The thalamus as a gate of access for sensory information to reach the cerebral cortex (18–20) is considered to play a crucial role in the pathophysiology of psychosis (21–23) as well as of bipolar disorder, particularly with a history of psychosis (24). Based on their heritability and state-independence, deficits in sensory gating have frequently been discussed as a potential neurophysiological endophenotype of psychosis. They have been shown in subjects at risk as well as in patients with first-episode and chronic psychosis, are present in both predominantly positive and negative symptom patients, and have also been found in unaffected relatives and in schizotypal personality disorder (25). However, this hypothesis needs further elucidation, as sensory gating deficits were significantly more pronounced in chronic psychosis compared to the at-risk state and first-episode psychosis suggesting that they might be a state marker of the expression of the disease rather than a state-independent marker of genetic risk for psychosis (26).

SEPs initially show a cortical low-frequency response occurring 20 ms after stimulation, i.e., the N20 (27), which is supposed to be generated by excitatory postsynaptic potentials in Brodman area 3b pyramidal cells (28). With high-pass filtering, an oscillatory burst of low-amplitude and high-frequency (600 Hz) wavelets superimposed on the N20 can be isolated (27, 29–33). These somatosensory high-frequency oscillations (HFOs) in SEP have been shown to play an important role in sensory information processing (34). Early and late HFOs, i.e., the wavelets before and after the peak latency of the initial N20, have been supposed to arise from separate generators since these two components differ in their responsiveness to various modulations, such as sleep-wake cycle or tactile interference (34, 35). The early part of the HFOs is presumably generated from action potentials of thalamocortical fibers (35–37). The generation of the late part of HFOs remains unclear as pyramidal “chattering” cells (38), cortical fast-spiking inhibitory interneurons (33) and thalamocortical relay cells (35) have been proposed as neuronal generators.

Like other early evoked potentials, i.e., occurring within 50 ms after stimulus application, SEPs are less susceptible to changes by uncontrollable factors such as attention than later potentials (17). Even if early evoked potentials have been less often studied than later ones, their underlying neurophysiology is better understood than that of the later potentials (14, 17). Therefore, altered SEPs might represent a robust indicator of vulnerability for schizophrenia and may offer the possibility of identifying potential patients before the onset of psychosis and of supporting prevention efforts.

The previously reported analysis of the baseline data of the present at-risk sample showed that alterations of early sensory filtering as measured by SEPs were observable also at the at-risk level (15). Subjects at risk for psychosis showed reduced low- and high-frequency source activity compared to subjects at risk for bipolar disorders and reduced high-frequency amplitudes compared to healthy controls. This is in line with the increasing body of evidence suggesting a sensory gating deficit in schizophrenia (39) and adds evidence to the assumption that specific sensory dysfunctions precede the onset of psychosis.

The aim of the present study was to explore the suitability of SEP as a neurophysiological marker. Therefore, we investigated the course of early sensory processing in help-seeking individuals being at risk to develop psychosis at the time of the baseline examination (15). Specifically, we distinguished between subjects remaining at risk at follow-up vs. subjects who converted to manifest psychosis during this time. Based on the progressive dynamics of structural and functional brain changes throughout the development of psychosis (40–42), the at-risk population was further subdivided into a high-risk (HR) and an ultra-high-risk (UHR) group. Low- and high-frequency components of SEPs measured at baseline and at follow-up 1 year later were analyzed in relationship to the clinical symptoms. Given that the previously reported differences in SEP measures at baseline were accentuated among cannabis non-users (15), we aimed to control specifically for the effect of cannabis in the present study, respectively, to explore SEP in cannabis-free subjects in case of an effect of cannabis in the whole group. We thought to find differences in SEP between converters and at-risk subjects as early as baseline, as it would be expected from a neurobiological marker.

Our research questions were therefore:

1. How do SEP parameters vary over time in subjects at risk with the HR group compared to the UHR group (respectively in cannabis-free subjects)?
2. How do SEP parameters vary over time in converters (retrospectively)?
3. Are these neurophysiological alterations (SEP variations over time) correlated with psychopathology (positive and negative symptoms at baseline)?
4. Are there differences between converters compared to subjects remaining at risk to develop psychosis at follow-up regarding early sensory filtering at baseline (retrospectively) and at follow-up (respectively among cannabis-free subjects)?

## Materials and Methods

### Subjects

Subjects at risk were recruited in the Canton of Zurich, Switzerland, within the framework of the “Zurich Program for Sustainable Development of Mental Health Services” (Zürcher Impulsprogramm zur nachhaltigen Entwicklung der Psychiatrie, i.e., ZInEP). For details regarding design, recruitment and sample of this prospective longitudinal multi-level approach on early recognition of psychoses, see Theodoridou et al. (43).

Psychiatrists, child and adolescent psychiatrists, psychologists, general practitioners, outreach clinics, counseling services, teachers, and affected persons or their family members could refer to one of the four regional early recognition units employing standardized criteria to identify persons at risk for psychosis and offering appropriate counseling. The assessment was conducted by trained and experienced psychiatrists or psychologists. SEP measurements were carried out in the neurophysiology lab at the central early recognition center.

After a complete description of the study to participants, written informed consent was obtained; in case of minors including the written informed consent of their parents. The study was approved by the regional ethics committee of the canton of Zurich and was performed in accordance with the Declaration of Helsinki. Subjects had to fulfill at least one of the following three inclusion criteria:

1. High-risk (HR) status for psychosis assessed by the adult (44) or children-youth (45) version of the Schizophrenia Proneness Interview (SPI-A/SPI-CY), with at least one cognitive-perceptive (COPER) basic symptom or at least two cognitive disturbances (COGDIS) basic symptoms.
2. Ultra-high-risk (UHR) status for psychosis as rated by the Structured Interview for Prodromal Syndromes (SIPS) (6) with at least one attenuated psychotic symptom, or at least one brief limited intermittent psychotic symptom, or a positive state-trait criterion (reduction in global assessment of functioning of >30% in the past year, plus either schizotypal personality disorder or first degree relative with psychosis).

The division into high-risk (HR) and ultra-high-risk (UHR) subjects within the psychosis-risk group was made with the aim to distinguish between subjects with a more general risk (HR, only COPER and/or COGDIS) and subjects with imminent risk [UHR, only UHR (= 15%) and UHR plus COPER and/or COGDIS (= 85%)] of transition to manifest psychosis (5, 46). In addition to the scales for early recognition, the Positive and Negative Syndrome Scale (PANSS) (47) was conducted to measure also the more pronounced psychotic symptoms. Exclusion criteria for study participation were manifest schizophrenic, substance-induced, or organic psychosis or bipolar disorder, current substance or alcohol dependence, as assessed by the Mini-International Neuropsychiatric Interview (MINI) (48); age below 13 or above 35 years; or low intellectual abilities with IQ < 80. Transition to psychosis was determined according to ICD-10 criteria.

Participants completed the SEP recordings shortly after study inclusion (t0, baseline assessment) and after 1 year (mean 12.9 months, *SD* = 2.5 months; t2, follow-up assessment). At baseline, SEP data were available from 155 subjects fulfilling inclusion criteria for HR and UHR groups. For comparison, 50 healthy controls (HC), matched regarding age and gender rates to the whole at-risk group, were enrolled in the study, 45 of them with available HF-SEPs. For socio-demographic details, see Hagenmuller et al. (15). The presence of any mental illness in the HC group was excluded using the MINI. All participants underwent a structural MRI at baseline prior to testing in order to exclude any brain abnormalities. At follow-up, SEP recordings were obtained from 54 subjects fulfilling inclusion criteria for HR or UHR groups at baseline. Due to project-specific issues, a follow-up assessment of the HC group could not be performed. Values in the HC group at baseline were taken as reference values.

Subjects classified as HR or UHR at baseline who converted to psychosis during the follow-up period (HR *n* = 1, UHR *n* = 7) were classified as the CONV group. Subjects classified as HR and UHR at baseline who did not convert still formed the HR resp. UHR groups (together termed the RISK group). Among the individuals from the HR, the UHR and the CONV groups, 37% at baseline and 46% at follow-up were receiving psychotropic medication, essentially antipsychotic and/or anti-depressive medication. The HC group did not differ statistically from the RISK group regarding age, sex and handedness. Specifically, we looked at subjects from the RISK group reporting no cannabis use at baseline, in contrast to cannabis users, i.e., subjects reporting frequent cannabis use (several times per week, weekly, and monthly). Subjects from the RISK group reporting rare use (less frequently than monthly) were not included in the cannabis related analysis. Individuals from the HC group reporting any cannabis use (*n* = 10) were excluded from the analysis. For details see Table 1.

**Table 1:** Descriptive characteristics of the subjects from the high-risk (HR), the ultra-high-risk (UHR) for schizophrenia and the converter (CONV) groups included in the follow-up analysis compared to the healthy control (HC) group.

		**RISK**			
	**HC**	**HR**	**UHR**	**CONV**	
*N*	35	19	27	8	
Age at baseline, years (SD)	20.8 (5.6)	23.4 (5.5)	18.8 (5.7)	18.8 (3.7)	
Gender (F:M)	16:19	9:10	11:16	4:4	
SIPS positive symptoms, WA^c^ (M, SD)					*Test statistics (group)*
- at baseline		5.3 (1.06, 0.70)	10.4 (2.07, 0.67)	9.5 (1.89, 0.85)	*F(2, 49)* = *10.38, p < 0.001*
- at follow-up		4.1 (0.81, 0.75)	5.1 (1.01, 0.64)	6.9 (1.37, 1.13)	*F(2, 42)* = *1.62, p = 0.210*
*Test statistics*		*t(15) = 1.74, p = 0.102*	*t(23)* = 6.29, *p < 0.001*	*t(6) = 0.85, p = 0.427*	
SIPS negative symptoms, WA^c^ (M, SD)					
- at baseline		13.1 (2.18, 0.89)	13.1 2.18 (1.22)	17.3 (2.88, 1.08)	*F(2, 49)* = *0.93, p = 0.401*
- at follow-up		6.5 (1.08, 0.95)	7.4 (1.23, 1.08)	11.0 (1.83, 1.82)	*F(2, 42)* = *0.46, p = 0.635*
*Test statistics*		*t(15) = 3.65, p = 0.002*	*t(23) = 3.05, p = 0.006*	*t(6) = 1.90, p = 0.107*	
Anti-depressive med. (*n*)	–				
- at baseline		5	5	1	
- at follow-up		7	7	3	
Antipsychotic med. (*n*, CPZe^a^)	–				
- at baseline		4 (85.2)	5 (104.2)	5 (230.3)	
- at follow-up		3 (166.9)	8 (117.8)	5 (275.1)	
Cannabis use at baseline					
yes/no (missings or not included)	0/35	8/19 (27)		1/7^b^	

a *Antipsychotic medication status is given in chlorpromazine equivalent (CPZe) dosage (49)*.

b *Cannabis analysis was not performed in the CONV group*.

c *WA, weighted average = mean scores × number of items (5 positive, 6 negative), M, mean; SD, standard deviation*.

*t- and p-values are given in italic type*.

### SEP Recording

Subjects were requested to sit in a comfortable chair with their eyes open, in a sound-attenuated laboratory room. They were instructed to relax and to avoid movements throughout the stimulus presentation sequence and the recording. Electrical transcutaneous stimulation was performed with two electrodes over the median nerve on the wrist of the dominant hand. Single constant-current square wave pulses of a duration of 0.2 s were delivered with an intensity of 4 mA above individual motor threshold (max. 20 mA) and a stimulus rate of 6 Hz during 12 min. To preserve a stable level of vigilance during stimulus presentation, participants were asked to watch a “Mr. Bean” movie without sound. EEG data were recorded using a BrainAmp amplifier and the Brain Vision Recorder software (both Brain Products GmbH, Munich, Germany). Electrodes were applied to the scalp using carefully positioned nylon caps (BrainCap with 32 channels, Easycap, Herrsching-Breitbrunn, Germany) in accordance with the international 10/20 system. Scalp electrode impedances were kept below 10 kΩ. EEG channels were referenced to FCz. Data were collected with a sampling rate of 2,500 Hz.

### SEP Data Analysis

**Figure 1:**
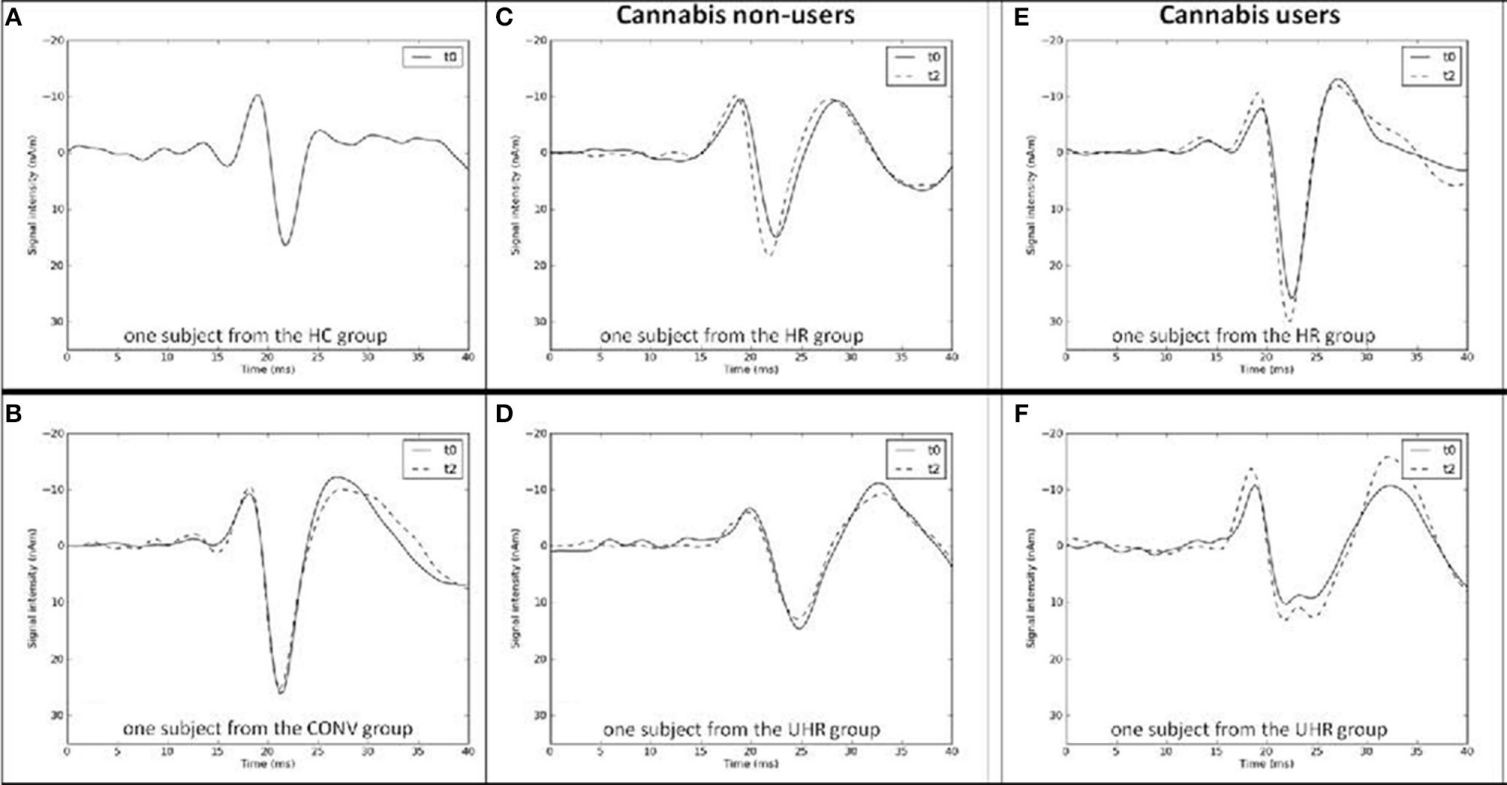
Examples of N20 curves at baseline (t0) and at follow-up (t2) from the **(A)** healthy control (HC), **(B)** converter (CONV), **(C)** high-risk (HR) cannabis non-users, **(D)** ultra-high-risk (UHR) cannabis non-users, **(E)** high-risk (HR) cannabis users, and **(F)** ultra-high-risk (UHR) cannabis users groups.

Using the Brain Electrical Source Analysis (BESA 5.1.8: MEGIS, Munich, Germany; www.besa.de) software, dipole source analysis was computed individually for each subject with at least 4,000 artifact-free sweeps over a period of 100 ms, from 20 ms before to 80 ms after the stimulation. Single dipole sources were fitted for each subject for a time period between 14 and 24 ms. An approach with one dipole was considered to be sufficient for demonstrating differences in signal composition between the subgroups, although an optimal source configuration would include at least three dipoles (14). This is in accordance with other studies, such as Norra et al. (50) or Waberski et al. (51). The resulting dipole waveform was digitally filtered with:

1. A low-pass filter of 450 Hz (12 dB/octave slope, zero phase shift) and a high-pass filter of 40 Hz (12 dB/octave slope, zero phase shift) to determine latency and strength of the low-frequency activity as estimated by dipole source analysis. The strength of the low-frequency activity source was determined semi-automatically as the absolute value of the minimum of the source waveform between 14 and 24 ms (N20). See Figures 1A–F for examples.
2. A low-pass filter turned off and a high-pass filter of 450 Hz (12 dB/octave slope, zero phase shift) to extract HFOs. Latencies of the negative oscillatory maxima and maximum peak-to-peak amplitudes were measured—peaking before the maximum of N20 for early HFO components, resp. after the N20 maximum for the late HFO components (52).

All peaks were plotted with the software Python (Python Language Website, http://www.python.org) and inspected visually.

### Statistical Analysis

The Kolmogorov-Smirnov test revealed that data were normally distributed (all *p* = n.s.). As the Levene test revealed homogeneity of most variances, parametrical tests were used.

1. Regarding the HR, UHR, and CONV groups over time, longitudinal data of interest (SEP parameters) were analyzed with paired *t*-tests and repeated-measures ANOVA with the within factor “time” and the between factor “cannabis use,” and “age” (in years) and “medication” (yes/no) at baseline as covariates. For clarity, only significant effects of covariates were mentioned. For the cannabis-related analysis, because of the small group size of cannabis users, we grouped the HR and the UHR groups (without CONV) into one RISK group. Significant effects of cannabis led to further analysis among cannabis non-users. To further explore changes over time, within-subject indices of N20 strengths were obtained by subtracting values at baseline from values at follow-up (N20 diff), with a negative result indicating a decrease in N20 strength (t0 > t2).
2. Correlation coefficients between SEP variations over time and clinical psychopathology were calculated with Pearson's *r*. The psychopathological data included SIPS positive and negative symptoms at baseline.
3. With regard to the HR, UHR, CONV, and HC groups at follow-up, cross-sectional group comparisons were performed with Student's *t*-test, ANOVA (N20 strength as dependent variable) or MANOVA (peak-to-peak amplitudes of HFO early and late parts resp. latencies of N20, HFO early and late parts as dependent variables). Age was entered as covariate (significant effects mentioned). Because the HC group was medication-free, the covariate medication was removed from the cross-sectional analyses including the HC group. In order to reduce possible errors due to multiple testing, simple contrasts were performed to look at the difference of the CONV group vs. the other groups at baseline and at follow-up.

Statistical significance was taken as *p* < 0.05. Based on the small sample sizes, effect sizes were provided for the group comparisons (Cohen's *d*, with *d* = 0.3 indicating a small, *d* = 0.5 a medium and *d* = 0.8 a large effect). SEP parameters were standardized by computing *z*-values based on the SEP values of the HC group at baseline. All statistical tests were performed using the IBM SPSS Statistics for Windows, Version 20.0 package (IBM Corp., Armonk, NY).

## Results

### Variation of SEP Parameters in the HR, UHR, and CONV Groups From Baseline to Follow-Up

SEP parameters of the HR and UHR groups at baseline and follow-up are given in Table 2. Within the HR and UHR group (without CONV), repeated measures ANOVA revealed no differences in all SEP parameters between baseline and follow-up, but a significant interaction of measurement time and cannabis use on the N20 strength [*F*_(1,23)_ = 8.51, *p* = 0.008]. In particular, cannabis non-users differed from cannabis users regarding the time-dependent evolution of the N20 source activity.

**Table 2:** SEP parameters for the high-risk (HR), the ultra-high-risk (UHR), and the converter (CONV) groups compared to the baseline values of the healthy control (HC) group, given in mean (SD).

				**RISK**			***Test statistics^a^***	
			**HC *N* = 35**	**HR *N* = 19**	**UHR *N* = 27**	**CONV *N* = 8**	***Group***	***Cannabis***
N20	Strength (nAm)	Baseline	10.54 (4.4)	8.65 (2.8)	8.54 (3.8)	10.81 (3.6)	*F*_(3, 62)_ = 1.98, *p = 0*.126	*F*_(1, 62)_ = 2.29, *p* = 0.135
		Follow-up	–	8.60 (3.5)	8.43 (3.6)	11.97 (3.1)	***F*_(3, 62)_ = 3.09, *p* = 0.033**	***F*_(1, 62)_ = 7.04, *p* = 0.010**
	*Test statistics*^b^	*time*		*F*_(1, 23)_ = 2.12, *p* = 0.159		*t*(7) = −1.59, *p* = 0.157		
		time^*^cannabis		***F*_(1, 23)_ = 8.51, *p* = 0.008**				
	Latency (ms)	Baseline	18.86 (1.5)	19.73 (0.8)	19.39 (1.1)	18.70 (2.4)	*F*_(3, 57)_ = 1.91, *p* = 0.139	*F*_(1, 57)_ = 1.02, *p* = 0.318
		Follow-up	–	19.37 (1.3)	19.89 (1.2)	18.95 (1.4)	*F*_(3, 53)_ = 1.35, *p* = 0.270	*F*_(1, 53)_ = 0.04, *p* = 0.949
	*Test statistics*^b^	*Time*		*F*_(1, 23)_ = 0.30, *p =* 0.591		*t*(7) = −0.22, *p* = 0.832		
		Time^*^cannabis		*F*_(1, 23)_ = 0.20, *p =* 0.656				
HFO early part	Strength (nAm)	Baseline	1.65 (1.0)	1.17 (0.6)	1.30 (0.64)	1.09 (0.4)	*F_(3, 57)_ = 2.28, p = 0.089*	*F*_(1, 57)_ = 1.70, *p* = 0.197
			–	1.12 (0.4)	1.45 (0.63)	1.47 (0.5)	*F_(3, 53)_ = 0.83, p = 0.485*	*F*_(1, 53)_ = 0.03, *p* = 0.956
	*Test statistics*^b^	*Time*		*F*_(1, 15)_ = 1.05, *p* = 0.321		***t*(5) = −2.50, *p* = 0.055**		
		Time^*^cannabis		*F*_(1, 15)_ = 0.33, *p* = 0.572				
	Latency (ms)	Baseline	17.72 (1.6)	18.33 (1.0)	18.21 (1.2)	17.38 (1.6)	*F*_(3, 57)_ = 1.70, *p* = 0.178	*F*_(1, 57)_ = 0.74, *p* = 0.392
		Follow-up	–	18.34 (0.8)	18.51 (1.0)	18.11 (0.7)	*F*_(3, 53)_ = 1.63, *p* = 0.194	*F*_(1, 53)_ = 0.22, *p* = 0.642
	*Test statistics*^b^	*Time*		*F*_(1, 15)_ = 0.59, *p* = 0.456		*t*(5) = −2.01, *p* = 0.101		
		Time^*^cannabis		*F*_(1, 15)_ = 3.36, *p* = 0.087				
HFO late part	Strength (nAm)	Baseline	1.66 (0.98)	1.07 (0.6)	1.25 (0.61)	1.04 (0.3)	***F*_(3, 57)_ = 2.88, *p* = 0.044**	*F*_(1, 57)_ = 2.90, *p* = 0.094
		Follow-up	–	1.12 (0.4)	1.19 (0.59)	1.40 (0.3)	*F*_(3, 53)_ = 1.12, *p* = 0.351	*F*_(1, 53)_ = 0.00, *p* = 0.994
	*Test statistics*^b^	*Time*		*F*_(1, 20)_ = 0.52, *p* = 0.480		***t*(6) = −2.61, *p* = 0.040**		
		Time^*^cannabis		*F*_(1, 20)_ = 1.56, *p* = 0.227				
	Latency (ms)	Baseline	19.69 (1.7)	19.03 (1.7)	20.47 (1.4)	19.94 (1.0)	*F_(3, 57)_ = 2.27, p = 0.090*	*F*_(1, 57)_ = 0.35, *p* = 0.559
		Follow-up	–	20.42 (0.6)	20.89 (1.5)	20.00 (1.3)	*F_(3, 53)_ = 2.19, p = 0.100*	*F*_(1, 53)_ = 0.14, *p* = 0.907
	*Test statistics*^b^	*Time*		*F*_(1, 20)_ = 0.80, *p* = 0.391		*t*(7) = −0.92, *p* = 0.395		
		Time^*^cannabis		*F*_(1, 23)_ = 0.48, *p* = 0.499				

a *ANOVA, with covariate age, baseline values from HC group included in comparison*.

b *Repeated measures ANOVA, with covariates age and medication*.

*Statistical significances and tendencies are given in bold type*.

The N20 strength of cannabis non-users at risk for psychosis seemed to decrease over time, while the N20 strength of cannabis users seemed to increase. The mean N20 strength of cannabis non-users was lower at follow-up than at baseline, but this difference did not reach significance [*t*(18) = 1.58, *p* = 0.131]. Moreover, cannabis non-users showed a significantly weaker mean N20 strength at follow-up compared to the HC group [*t*(52) = 2.96, *p* = 0.005, *d* = 0.821].

**Figure 2:**
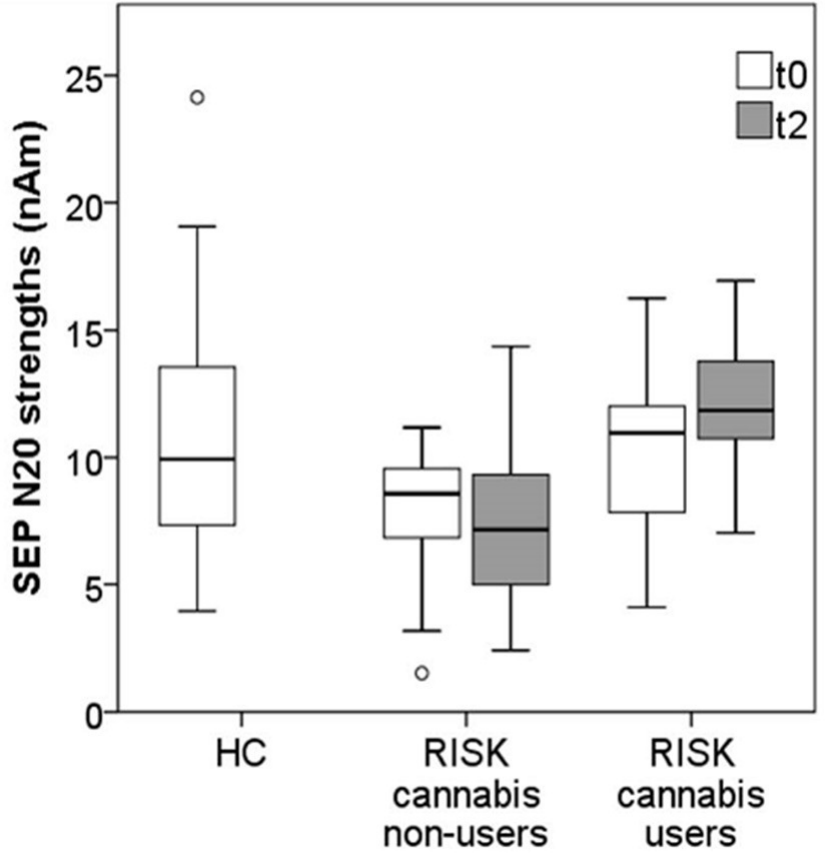
Mean N20 strengths in cannabis non-users (*n* = 19) vs. cannabis users (*n* = 8) from the RISK group at baseline (t0) and at follow-up (t2), compared to the reference values from the HC group (*n* = 35).

On the other hand, the mean N20 strength of cannabis users was significantly higher at follow-up than at baseline [*t*(7) = −2.59, *p* = 0.036, *d* = 1.958]. Interestingly, the mean N20 strength of cannabis users did not, at follow-up, differ significantly from the HC group [*t*(41) = −0.65, *p* = 0.523], see Figure 2.

Contrary to the observations in the HR and UHR groups, SEP parameters of the CONV group seemed to increase over time. In particular, paired *t*-tests revealed that the CONV group showed a significantly stronger HFO late part at follow-up compared to baseline [*t*(6) = 2.61, *p* = 0.040, *d* = 2.131, see Table 2].

### Relationship Between SEP Parameters, Psychopathology, and Antipsychotic Medication

With regard to the psychopathological data, there were no differences in SIPS scores between cannabis non-users and cannabis users neither at baseline [positive, negative, disorganized, and general symptoms, *t*(28) < 0.7, *p* > 0.54] nor at follow-up [*t*(25) < 1.46, *p* > 0.16]. Moreover, there were no differences in antipsychotic medication between cannabis non-users and cannabis users neither at baseline [chlorpromazine equivalent [CPZe), *t*(28) < 0.72, *p* > 0.48] nor at follow-up [*t*(25) < 1.6, *p* > 0.12].

**Figure 3:**
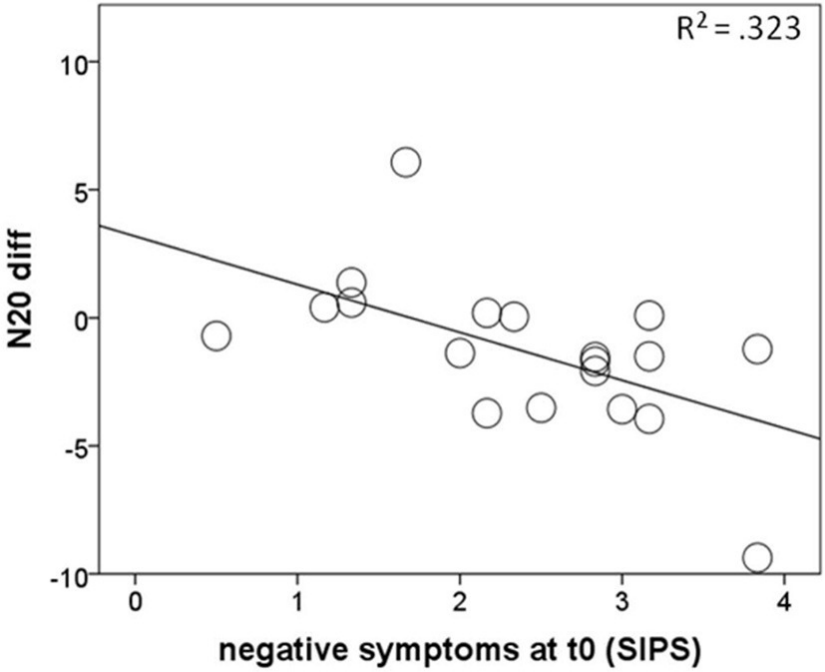
Correlations of negative symptoms at baseline with the amount of N20 strength decrease over time (N20 diff) in cannabis non-users from the RISK group, with negative values representing smaller values at follow-up than at baseline.

To further explore the decrease of the N20 strength in cannabis non-users, correlations between within-subject indices of the evolution of the N20 strengths (N20 diff) over time and the extent of the positive and negative symptoms at baseline were calculated. N20 diff correlated negatively with SIPS negative symptoms at baseline (*r* = −0.57, *p* = 0.011), indicating higher scores on the negative symptoms subscale of the SIPS at baseline being associated with a greater decrease of N20 strengths over time (Figure 3). On the other hand, there was no relationship between the N20 decrease and SIPS positive symptoms at baseline.

Given the potential impact of antipsychotic medication on the neurophysiological data, additional correlational analyses on the relationship between SEP parameters and CPZe were performed. However, there was no relationship between SEP parameters and antipsychotic medication neither at baseline (*r* < 0.31, *p* > 0.30) nor at follow-up (*r* < −0.18, *p* > 0.19).

### SEP Parameters at Baseline

At baseline, there was a significant main effect of group on the strengths of HFO late part [*F*_(3, 57)_ = 2.88, *p* = 0.044], but not on the strengths of the N20 nor HFO early part (see Table 2). Planned contrasts revealed that, at baseline, the CONV group showed a statistical trend toward lower amplitudes of HFO late part [0.62, 95%CI (– 0.12, 1.36), *p* = 0.095, *d* = 0.85] compared to the HC group. On the other hand, the CONV group did not differ from the HR nor UHR groups. Cannabis use had no significant effect on the SEP parameters at baseline.

### SEP Parameters at Follow-Up

At follow-up, there was a significant main effect of group on the N20 strength [*F*_(3,62)_ = 3.09, *p* = 0.033, see Table 2]. Planned contrasts revealed that the CONV group had significantly stronger N20 intensity compared to the UHR group [4.96, 95%CI (0.47, 9.45), *p* = 0.031, *d* = 1.057]. There was also a significant main effect of cannabis [*F*_(1,62)_ = 7.04, *p* = 0.010], so that we repeated group comparisons within the cannabis non-users subgroup.

Among cannabis non-users, ANOVA showed significant differences between groups (HC, HR, UHR, CONV) regarding N20 source strengths [*F*_(3,56)_ = 3.22, *p* = 0.029]. Planned contrasts revealed that the UHR group had significantly weaker N20 strengths [−3.89, 95%CI (−6.76, 1.02), *p* = 0.009, *d* = 1.01] compared to the HC group. In contrast, the HR and the CONV groups did not significantly differ from the HC group regarding these parameters.

**Figure 4:**
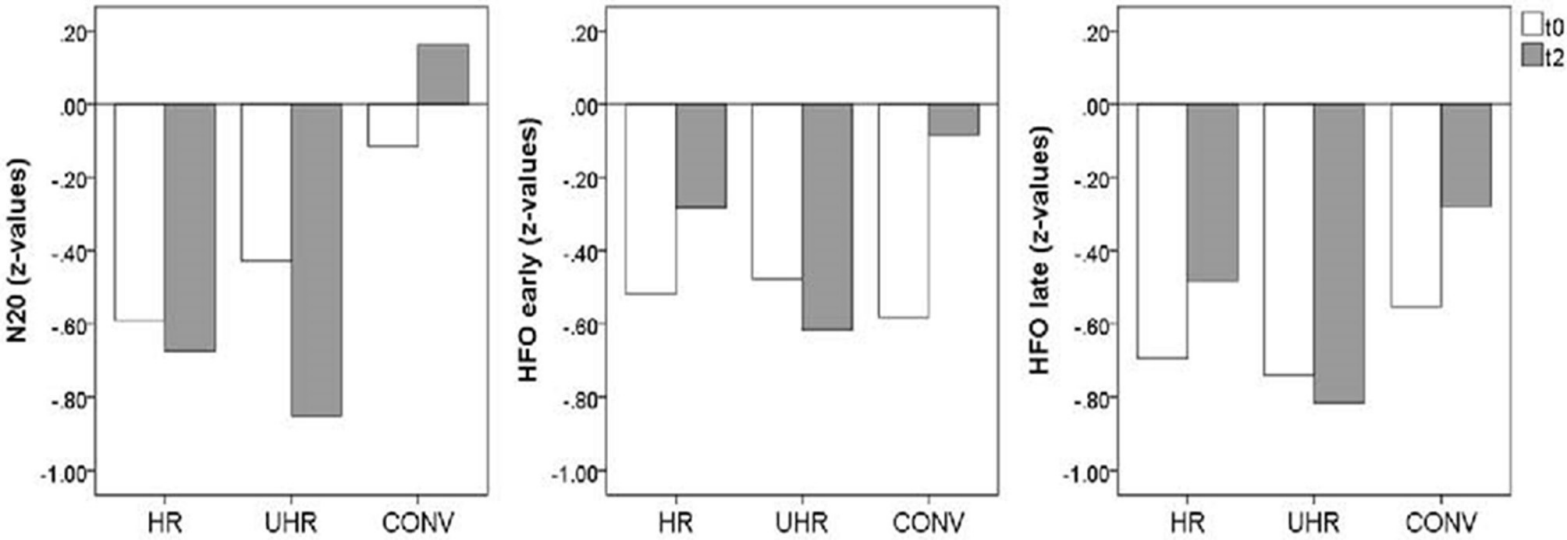
Mean SEP amplitudes (*z*-values) for the cannabis non-user subjects from the high-risk (HR, *n* = 10), ultra high-risk (UHR, *n* = 9), and converter (CONV, *n* = 7) groups at baseline (t0) and follow-up (t2).

Removing the HC group for an exploration of the differences between at-risk subjects vs. converters at follow-up, ANOVA showed significant differences among cannabis non-users regarding N20 source strengths [*F*_(2,21)_ = 4.87, *p* = 0.018] and amplitudes of the late part of HFOs [*F*_(2, 13)_ = 5.31, *p* = 0.021]. Moreover, there was a trend regarding the amplitudes of the early part of HFOs [*F*_(2,13)_ = 3.56, *p* = 0.058]. Planned contrasts revealed that the CONV group had stronger N20 source activity compared to the HR [−3.49, 95%CI (−6.88, −0.09), *p* = 0.045, *d* = 1.409] and the UHR group [−4.63, 95%CI (−7.74, −1.51), *p* = 0.006, *d* = 1.739], as well as greater early HFO amplitudes [−0.68, 95%CI (−1.30, −0.07), *p* = 0.032, *d* = 0.987] and greater late HFO amplitudes compared to the UHR group [−0.62, 95%CI (−1.03, −0.21), *p* = 0.006, *d* = 1.434]. In addition, there was a statistical trend for greater HFO amplitudes in the CONV group compared to the HR group [−0.63, 95%CI (−1.28, 0.015), *p* = 0.055, *d* = 0.544], as shown in Figure 4. Finally, there was a significant effect of medication on late HFO amplitudes [*F*_(1,13)_ = 9.98, *p* = 0.008].

## Discussion

In this study, low and high frequency SEP data were collected at baseline and 1 year later at follow-up in 54 subjects who were at risk for developing psychosis at time of baseline examination. This study investigated the course of early sensory filtering preceding the (putative) onset of psychosis. To our knowledge, this is the first study investigating SEPs in a longitudinal design in populations at risk for developing psychosis to date. Based on the baseline findings (15), we conducted our analysis regarding cannabis, respectively, we focused specifically on cannabis-free subjects.

The longitudinal development of the N20 strength depended on cannabis use. The N20 strengths of cannabis non-users seemed to decrease over time. SIPS negative symptoms were associated with this decrease, i.e., more negative symptoms at baseline were associated with a greater decrease of N20 strength over time. Additionally, we found that cannabis-free subjects at risk—and specifically at ultra-high-risk for psychosis—had weaker N20 strengths at follow-up, compared to the HC group. These results are in line with our baseline results (15) and previous reports on decreased early evoked responses in the at-risk state for psychosis (26). Amplitude reductions in the context of reduced sensory gating were interpreted as a reduced sensory registration (26). Sensory registration can be characterized by high sensory thresholds, i.e., subjects with low sensory registration pattern may have more difficulties than others to notice sensory stimuli (53). These observed amplitude reductions in subjects at risk suggest that sensory registration deficits may precede the putative onset of psychosis. Furthermore, it has been proposed that gating deficits are mainly associated with the negative symptoms of schizophrenia (54–58). Because they are related to decreased functional outcomes and refractory to common psychopharmacological treatments (59), negative symptoms seem to contribute to poorer quality of life early in the course of schizophrenia (60, 61) and may even represent themselves a vulnerability for the risk of developing psychosis (62). Moreover, the thalamus that is supposed to play a key role in somatosensory gating might be specifically associated with negative symptoms in schizophrenia (63–66). This association was also suggested in adolescents at ultra-high-risk for psychosis in a recent study in the context of increased sleep dysfunction (67). Negative symptoms have been described as emerging from a failure of learning during social interactions due to abnormal synaptic plasticity, the resulting experience of an unpredictable world being likely to trigger social withdrawal and apathy (68). In this line, Arnfred and Chen (69) have reported an association of early somatosensory information processing deficit and higher social anhedonia scores. They have hypothesized that a lack of somatosensory readiness degrading the fine-tuning of bodily attention would lead to aversive experiences of unpreparedness, secondarily followed by withdrawal and anxiety (69).

The biological basis of sensory registration deficits in subjects at risk as well as in patients with manifest psychosis is still not clear. Alterations in early evoked potentials, such as SEPs, are typically referred to disturbances of afferent sensory tracts and impaired thalamic filtering. However, abnormal functional interactions between the cortex and thalamus should be considered as well. In this context, several imaging studies reported characteristic dysfunctions of thalamocortical networks in schizophrenia that are closely associated with clinical and cognitive symptoms (70). With regard to early sensory information processing, Woodward et al. (71) could demonstrate, by using resting-state fMRI, marked differences in thalamocortical connectivity in patients with schizophrenia compared to healthy controls, with increased motor/somatosensory-thalamic connectivity and reduced pre-frontal-thalamic connectivity. Interestingly, this pattern of thalamocortical dysconnectivity was not only present in chronic schizophrenia but also in early stages of psychosis (72), presumably due to atypical brain maturation and/or distinct genetic factors (73). Moreover, it cannot be ruled out that abnormal functional interactions within specific cortical networks may be involved in the deficits in sensory information processing as described in our analysis. In particular, disturbed functional connectivity and organization within the somatosensory and sensorimotor cortex in schizophrenia have been reported by several neurophysiological studies (74–76). Further research is required in order to elucidate the etiological significance.

In our sample, contrary to subjects at risk, the group of converters showed no changes in negative symptoms between baseline and follow-up. Our retrospective analysis showed that, at baseline, the group of converters differed from the control group regarding amplitudes of HFOs, as did at-risk subjects in our baseline study (15). However, at baseline, the group of subjects with future transition to psychosis did not differ from other subjects remaining at risk at follow-up. These results are in accordance with other studies (26, 77) which found deficits in sensory registration in subjects at risk compared to controls but did not find any differences between at-risk with and without conversion. Consequently, it has been suggested that these deficits in sensory registration may not represent a marker for a genetic risk for psychosis but rather be influenced by state-dependent factors (26). Alternatively, it cannot be excluded that putative converters are confounded within the at-risk groups and that a longer follow-up period would have influenced these results by providing more transitions to psychosis. Furthermore, due to different treatment regimens between baseline and follow-up, some conversions have possibly been prevented by treatment. Finally, a lack of statistical power due to the very small sample size of the converter group cannot be excluded.

At follow up, according to our expectations, we found differences in SEP parameters between the group of converters and the subjects remaining at risk, but these differences turned out to be somewhat counterintuitive. Converters showed stronger SEP parameters than at-risk subjects but did not differ from the HC group. It is not clear how this should be interpreted. In contrast to the present findings, previous studies on SEP in patients with chronic schizophrenia reported higher N20 mean amplitudes (16, 17, 50) and later HFO than controls (50). A higher N20 amplitude was interpreted as an impairment of thalamic filtering of the afferent sensory information to the cortex (50). More generally, the present findings stand in contradiction to the extensive literature reporting decreased evoked responses in patients with schizophrenia, particularly decreased early responses such as N100 and P50 (56, 78). However, Valkonen-Korhonen et al. (79) also found no difference in early attention-independent automatic processing (100 ms after stimulus) in never-medicated first episode patients compared to healthy controls and reported that otherwise observed deviances appeared only in attention-dependent processing after about 250 ms. Furthermore, Salisbury et al. (80) reported deviant mismatch negativity (MMN) in patients with chronic schizophrenia that were not present at first hospitalization as well as opposite associations of pattern of symptoms with MMN amplitudes in first episode patients (smaller amplitudes) vs. in chronic patients (larger amplitudes). Taken together, a transition to psychosis resp. a first episode may represent an interstage between a reduced sensory registration from the at-risk state and a gating deficit in the chronic state. Our longitudinal analyses accounted for potential medication impact, but we did not differentiate the effects of antidepressants vs. antipsychotics because of the small sample size and the cross-sectional analyses. We omitted the covariate medication in the cross-sectional analyses because it was closely related to the group factor (the HC group was medication-free). Consequently, it cannot be ruled out that medication has an effect on praxis level on all SEP parameters, particularly in the converter group that showed the highest dosage. Moreover, the fact that antipsychotic drugs are not equally effective for all patients with psychosis contribute to the assumption that heterogeneity of different underlying disease mechanisms may lead to a psychopathology with similar clinical presentation. For example, different neuromodulatory dysregulations—such as dopaminergic, cholinergic, or serotonergic dysregulation of NMDA-mediated synaptic plasticity—are hypothesized to lead to the development of psychotic symptoms (68). Therefore, our sample of subjects with resp. without transition to psychosis would represent a heterogeneous group (81) with regard to underlying synaptic plasticity. Furthermore, symptoms of depression, anxiety and psychosis often overlap in subjects at high risk of psychosis (82). Besides this, different patterns of brain structure alteration were suggested in subjects in at-risk state with genetic risk vs. with attenuated psychotic symptoms, the subgroup with genetic risk showing more similarities of morphology to patients with a first episode psychosis, one of them affecting the right thalamus (83). Given that most of the at-risk subjects do not convert to manifest psychosis, the biological significance of the decreased N20 strengths in our at-risk groups remains unclear and needs further replication.

Contrary to the decrease in N20 strengths over time in the cannabis non-users group, the N20 strengths of cannabis users seemed to increase over time, being similar at follow-up to the reference values from the HC group. As it was hypothesized previously (11, 84), cannabis may produce transient symptoms and deficits resembling those seen in schizophrenia, thereby increasing clinical false positive ratings. Additionally, a superior cognitive profile in schizophrenia-spectrum disorders with concomitant cannabis use has been reported (85–87), but this was suggested to be associated with better social skills (in order to be able to acquire and sustain drug habit) rather than to reflect a neuroprotective effect of THC (84, 88). In this line, Koenders et al. (89) have provided the possible explanation that non-users are a group in whom psychosis may develop without the extra-risk factor of cannabis use, i.e., that patients who use cannabis may be less intrinsically vulnerable for psychosis than non-users.

Our study has methodological limitations. First, the group of converters was very small, so that statistic power may be limited and results in this group have to be interpreted with caution. Based on the heuristic and exploratory nature of the study, we did not consider a Bonferroni correction for multiple testing and recommend to replicate the findings in future studies with greater sample sizes. Moreover, the HC group has not been screened for prodromal symptoms at baseline by standardized measures, such as SPI-A or SIPS. Therefore, the validity of the results may be limited as the potential influence of undiscovered pre-psychotic syndromes at the low-intensity level cannot be completely ruled out. Then, cannabis analysis is limited because many data is missing, we relied on self-reports and did not perform urinary screening as recommended in the literature (90) and we did not differentiate between acute and chronic effects of cannabis (91). However, an analysis of cannabis effects was not the aim of this paper. On the other hand, the observed results might be confounded with an effect of alcohol and tobacco, as several subjects being at risk for psychosis may be prone to use these substances. At last, an examination of the HC group was performed only at baseline. We assumed that the time effect of 1 year on SEP parameters would be negligible in healthy participants. We are aware that small developmental changes in the somatosensory afferent pathway from spinal cord to thalamus seem to be complete by age 17 (92) and some of our participants were younger than this age. Increases in EP latency with age were reported (92). Then, the amplitude of the cortical primary response is known to follow a U-shape curve with aging, being high during adolescence, low during middle age and high again in old age (93–95). Additionally, a comparison of participants older than 60 years with participants in the mid-twenties suggested that the later part of HFOs is associated with aging (96). However, our groups were all in similar ages and statistical analysis did not show any effect of age. None the less, a follow-up examination of the HC group would have provided further insight. Finally, the presently reported method of dipole source analysis is not suitable for clinical practice. Further experimentation is needed to determine if a simple one-channel information would lead to comparable results.

In summary, the observed deficits in sensory registration may not represent a marker for a genetic risk for psychosis but rather reflect state-dependent factors, e.g., negative symptoms. These amplitude reductions in subjects at risk vs. the lack of differences between converters and controls resp. the amplitude increases in chronic schizophrenia previously reported in the literature might suggest that a transition to psychosis resp. a first episode may represent an interstage between a reduced sensory registration from the at-risk state and a gating deficit in the chronic state.

## Author Contributions

FH, KH, HH, AT, SW, WR, and WK designed the study and wrote the protocol. FH, KH, and PR managed the literature searches and analyses. FH undertook the statistical analysis. FH, KH, and PR wrote the first draft of the manuscript. All authors contributed to and have approved the final manuscript.

### Conflict of Interest Statement

The authors declare that the research was conducted in the absence of any commercial or financial relationships that could be construed as a potential conflict of interest.
